# Diabetic Microvascular Complications

**DOI:** 10.1155/2018/5683287

**Published:** 2018-05-28

**Authors:** I. Migdalis, L. Czupryniak, N. Lalic, R. D. Leslie, N. Papanas, P. Valensi

**Affiliations:** ^1^Second Medical Department and Diabetes Centre, NIMTS Hospital, 12 Monis Petraki, 11521 Athens, Greece; ^2^Department of Diabetology and Internal Medicine, Warsaw Medical University, Warsaw, Poland; ^3^Faculty of Medicine, University of Belgrade, Clinic for Endocrinology, Diabetes and Metabolic Diseases, CCS, Belgrade, Serbia; ^4^Department of Diabetes, Saint Bartholomew's Hospital, University of London and Blizard Institute, EC1A 7BE London, UK; ^5^Diabetic Foot Clinic-Diabetes Centre, Second Department of Internal Medicine, Democritus University of Thrace, 68100 Alexandroupolis, Greece; ^6^Department of Endocrinology, Diabetology and Nutrition, Jean Verdier Hospital, AP-HP, Paris Nord University, CRNH-IdF, CINFO, 93140 Bondy, France

Microvascular complications account for a substantial increase in morbidity and a considerable impairment in the quality of life in patients with diabetes mellitus (DM) [1–3]. The present special issue provides new research data in the field of diabetic microvascular complications.

T. Tönnies et al. in their paper entitled “Risk of Microvascular Complications and Macrovascular Risk Factors in Early-Onset Type 1 Diabetes after at Least 10 Years Duration: An Analysis of Three Population-Based Cross-Sectional Surveys in Germany between 2009 and 2016” examined patients with early-onset and long duration type 1 diabetes. They reported an increased risk of microvascular complications at 14 years after the diagnosis of type 1 DM. Obviously, there is a lot to learn and improve in our understanding of the evolution and prevention of complications in such patients.

P. Ruiz-Ocaña et al. in their paper “Decreased Retinal Thickness in Type 1 Diabetic Children with Signs of Nonproliferative Diabetic Retinopathy” evaluated retinal thickness in paediatric patients with type 1 DM in relation to nonproliferative diabetic retinopathy, glycaemic control, and DM duration. They observed decreased thickness and volumes of retina in the presence of nonproliferative diabetic retinopathy.

Y. Takamura et al. in their article “Direct Photocoagulation Guided by Merged Retinal Images for the Treatment of Focal Diabetic Macular Edema” introduced the new merged image-guided laser photocoagulation for the treatment of focal diabetic macular edema. They documented improvements in visual acuity and retinal thickness, as well as in the number of laser shots required and in the need for new treatment. These results are very promising.

D. Zhang et al. in their paper “Elevated Serum Total Bilirubin Concentrations are Negatively Associated with Diabetic Retinopathy among the Chinese Northeastern Population” showed a significant negative association between serum total bilirubin and diabetic retinopathy in Chinese patients. They suggested that total bilirubin might prove useful in the early detection of this complication.

M. Šimunović et al. studied the early development of cataract in children and adolescents with type 1 DM. In their work entitled “Cataract as Early Ocular Complication in Children and Adolescents with Type 1 Diabetes Mellitus,” they found a 0.7–3.4% prevalence of cataract in children and adolescents with T1DM. Cataract development frequently occurred in as the first sign of or during the first 6 months of diagnosis of type 1 DM.

In their paper entitled “Characterization of In Vivo Retinal Lesions of Diabetic Retinopathy Using Adaptive Optics Scanning Laser Ophthalmoscopy,” S. G. Karst et al. found that adaptive optics scanning laser ophthalmoscopy can provide high-resolution visualisation of diabetic retinopathy. An additional advantage was the assessment of retinal perfusion and the identification of small vascular lesions.

M. A. Ahmed et al. in their paper “Perspectives on Peripheral Neuropathy as a Consequence of Metformin-Induced Vitamin B12 Deficiency in T2DM” have summarised all contemporary results of the available and rather conflicting evidence on the relationship between vitamin B12 deficiency, the potential contribution of metformin therapy, and the development of diabetic peripheral neuropathy. There is a lot to appreciate, but, as the authors rightly point out, there is also a need for more appropriate design of further trials.

In their work “Susceptible and Prognostic Genetic Factors Associated with Diabetic Peripheral Neuropathy: A Comprehensive Literature Review,” L. B. L. Prabodha et al. have reviewed the role of the genetic component (susceptibility and prognostic factors, mutations, and polymorphisms) in the development of peripheral neuropathy in type 2 DM. Identification of common genetic variants along with additional gene expression studies may enable future gene-targeted therapies, and this is eagerly awaited.

T. Didangelos et al. in their paper “A Comparative Assessment of Cardiovascular Autonomic Reflex Testing and Cardiac ^123^I-Metaiodobenzylguanidine Imaging in Patients with Type 1 Diabetes Mellitus without Complications or Cardiovascular Risk Factors” compared the diagnostic performance of cardiovascular autonomic reflex tests with that of sympathetic innervation heart imaging with ^123^I metaiodobenzylguanidine for cardiac autonomic neuropathy in type 1 DM. The former yielded high sensitivity but low specificity, as compared with the latter, and it also depended on the duration of type 1 DM.

L. Yazdanpanah et al. in their paper “Incidence and Risk Factors of Diabetic Foot Ulcer: A Population-Based Diabetic Foot Cohort (ADFC Study)—Two-Year Follow-Up Study” found a 2.8% average annual incidence of diabetic foot ulcers in Iran. Risk factors of ulcers were prior ulcer/amputation, insulin therapy, male gender, neuropathy, and foot deformity.

A. Gatt et al. in their work entitled “Establishing Differences in Thermographic Patterns between the Various Complications in Diabetic Foot Disease” evaluated the utility of thermography in the identification of foot temperature patterns in patients with DM. Foot temperature was significantly higher in the presence of diabetic complications. Intriguingly, higher temperature was associated with increased likelihood of neuropathy and/or peripheral arterial disease. These results add to the accumulating data on the importance of temperature measurements in the diabetic foot [4].

In their article “Oxidative Stress, Apoptosis, and Mitochondrial Function in Diabetic Nephropathy,” S. Sifuentes-Franco et al. reviewed the current views on the pathogenesis of diabetic nephropathy. Chronic hyperglycaemia, oxidative stress, apoptosis, and mitochondrial dysfunction are key underlying factors. Improved insight into the precise role and the interplay of these factors is expected to offer more efficacious treatment options.

J. Labad et al. in their article “Limited Joint Mobility Progression in Type 1 Diabetes: A 15-Year Follow-Up Study” demonstrated significant reductions in the flexions of the 5th metacarpal and wrist joints at 15 years after the initial examination in patients with type 1 DM. This deterioration was more pronounced among patients with microalbuminuria.

Finally, M. J. Crespo et al. in their paper “Synergistic Effects of Dantrolene and Nimodipine on the Phenylephrine-Induced Contraction and ACh-Induced Relaxation in Aortic Rings from Diabetic Rats” examined streptozotocin-induced diabetic rats. They showed beneficial effects of dantrolene and nimodipine combination in decreasing arterial tone of their aortic rings.

There are abundant and useful new data from the research of diabetic microvascular complications. This ongoing research will enrich our understanding in the area and eventually contribute to improved therapeutic modalities in the near future [5].

*I. Migdalis* *L. Czupryniak* *N. Lalic* *R. D. Leslie* *N. Papanas* *P. Valensi*

## References

[1] Armstrong D. G., Boulton A. J. M., Bus S. A. (2017). Diabetic foot ulcers and their recurrence. *New England Journal of Medicine*.

[2] Migdalis I., Leslie D., Papanas N., Valensi P., Vlassara H. (2014). Diabetes mellitus. *International Journal of Endocrinology*.

[3] American Diabetes Association (2017). 10. Microvascular complications and foot care: standards of medical care in diabetes—2018. *Diabetes Care*.

[4] Sibbald R. G., Mufti A., Armstrong D. G. (2015). Infrared skin thermometry: an underutilized cost-effective tool for routine wound care practice and patient high-risk diabetic foot self-monitoring. *Advances in Skin & Wound Care*.

[5] Cardoso C. R. L., Leite N. C., Moram C. B. M., Salles G. F. (2018). Long-term visit-to-visit glycemic variability as predictor of micro- and macrovascular complications in patients with type 2 diabetes: The Rio de Janeiro Type 2 Diabetes Cohort Study. *Cardiovascular Diabetology*.

